# Determination of predictors associated with pain in non‑surgically treated adults with idiopathic scoliosis

**DOI:** 10.1186/s13018-024-04912-8

**Published:** 2024-07-16

**Authors:** Mehmet Yetiş, Nazım Tolgahan Yildiz, Mehmet Canli, Hikmet Kocaman, Hasan Yildirim, Halil Alkan, İrem Valamur

**Affiliations:** 1 Faculty of Medicine, Department of Orthopedics and Traumatology, Kırşehir Ahi Evran University, Kırşehir, Turkey; 2 Faculty of Health Sciences, Deparment of Physiotherapy and Rehabilitation, Karamanoglu Mehmetbey University, Karaman, Turkey; 3 School of Physical Therapy and Rehabilitation, Kırşehir Ahi Evran University, Kırşehir, Turkey; 4 Faculty of Kamil Özdağ Science, Department of Mathematics, Karamanoğlu Mehmetbey University, Karaman, Turkey; 5 Faculty of Health Science, Deparment of Physiotherapy and Rehabilitation, Muş Alparslan University, Muş, Turkey

**Keywords:** Scoliosis, Pain, Determinants, Relations

## Abstract

**Background:**

It is recognized that pain related to adult individuals with idiopathic scoliosis (IS) substantially impacts individuals’ daily activities and quality of life. The objective of this study was to identify the possible predictors of pain intensity in non‑surgically treated adults with IS.

**Methods:**

This cross-sectional study included 58 adults individuals with Lenke type 1 IS. Participants’ sociodemographic characteristics were recorded, and pain severity, curvature severity, trunk rotation angle, disability, spinal mobility, cosmetic deformity perception, and quality of life were assessed. Regression analyses with various models were performed to determine the predictors of pain severity and the best model was selected based on performance criteria.

**Results:**

Strong associations were found between pain severity with curvature severity, spinal mobility, trunk rotation angle, perception of cosmetic deformity, disability, and quality of life (*p* < 0.05). It was observed that Lasso regression was the best model based on the performance criteria considered. According to this model, the primary predictors of pain intensity in adult IS were determined as curvature severity, spinal mobility, trunk rotation angle, cosmetic deformity perception, back-related disability and quality of life, in order of importance.

**Conclusion:**

In accordance with the findings of this study, which examined for the first time the determinants of pain intensity in adult individuals with Lenke type 1 IS, we suggest that mentioned possible factors affecting and determining pain should be taken into consideration when establishing evaluation and treatment programs.

## Introduction

Idiopathic scoliosis (IS) is a complex three-dimensional deformity of the spine, defined as a lateral curvature of the spine greater than 10° of unknown cause. The deformity occurs during skeletal growth and affects approximately 1–4% of children [1]. Most children with IS are asymptomatic, but progressive curvatures need to be treated in order to change the natural course of the deformity [2]. Surgical treatment is recommended for curvatures exceeding 45°-50° in order to prevent progression of the curve after the skeleton matures and subsequent adverse effects in adulthood [3]. Exercise therapy, steroid injections, non-steroidal anti-inflammatory drugs, and the use of narcotics are among the non-surgical treatment methods for IS [4].

Adult spinal deformities (ASD) encompass a range of pathologies with various radiographic and clinical findings. Furthermore, the estimated prevalence in the general population of adults aged 60 years and older is approximately 60–70% [5]. Adult IS is one of these types of ASD and is characterized by progression of spinal deformity beginning in the adolescent years. Thus, adult IS typically has deformities in the coronal, sagittal, and axial planes and frequently involves both the thoracic and lumbar spines. This should not be confused with degenerative scoliosis, another common form of ASD, which occurs due to asymmetric aging of the intervertebral discs and facet joints [6].

Adults with chronic pain associated with IS with major curves that are not surgically corrected face many problems. It is reported that most adult individuals with IS suffer from chronic pain, and pain severity may related to age and degree of curvature [7]. Adult individuals with IS usually have back pain, leg pain, neurological deficit and claudication symptoms [8, 9]. Back pain is considered an expression of muscle fatigue or mechanical imbalance. Paravertebral back muscles, when unstable, overloaded, or strained, can induce significant pain and diminish their stabilizing capacity. This phenomenon may initiate a vicious cycle characterized by persistent, non-specific back pain and muscle spasm. However, the source of pain in adult individuals with IS is currently not completely clear [8].

In previous studies, it was concluded that conditions such as age, curve size, sleep disturbance, kinesiophobia, depression, and anxiety were associated with pain intensity in individuals with scoliosis [10–14]. For example, Fekete et al. [15] reported that age affected pain intensity in adolescent idiopathic scoliosis (AIS) and adult individuals with IS. In another study, Gremeaux et al. [16] reported that Cobb angle and trunk rotation angle (TRA) were associated with pain intensity in adult individuals with IS with low back pain. However, there are also some studies, minority of which indicate that pain intensity is not significantly correlated with age, skeletal maturity, and type of curvature [17].

To the best of the authors’ knowledge, no study in the literature has investigated the predictive factors influencing pain intensity in adult individuals with IS. Identifying the independent determinants of pain in adult individuals with IS could provide valuable guidance for clinicians in monitoring pain and developing assessment and treatment protocols. Therefore, the objective of this study was to identify the independent predictors of pain intensity in adult individuals with IS.

## Materials and methods

### Study design and ethical aspects

This cross-sectional study adhered to the STROBE guidelines, aimed at enhancing the reporting quality of observational epidemiological studies. Approval for the study protocol was granted by the Ethics Committee of Muş Alparslan University (Decision no: 6-2024/20). Prior to participation, all patients provided both written and verbal informed consent. The study was conducted in compliance with the ethical principles outlined in the Declaration of Helsinki.

### Participants

In this multicenter study conducted in Turkey, individuals who applied to the Orthopaedics and Traumatology Outpatient Clinics, were diagnosed with adult IS by a specialist physician, and met the inclusion criteria were included in the study. Inclusion criteria comprised Lenke type 1 adult individuals with IS with a thoracic Cobb angle > 30°, no history of surgical intervention, age over 18 years with a Risser stage of 5, and absence of conservative scoliosis treatment within the past year. The exclusion criteria encompassed patients with scoliosis types other than idiopathic (e.g., neuromuscular, syndromic, congenital, or secondary scoliosis), as well as those with other Lenke types of scoliosis (types 2–6), a history of prior spinal surgery or trauma, and diagnoses of rheumatic or neurological disorders. Demographic and clinical parameters were documented, and subsequent assessments were conducted.

### Assessment of pain intensity

Back pain intensity at the time of test completion among adult individuals with IS was evaluated utilizing the Visual Analogue Scale (VAS), which features a 10 cm linear scale extending from “no pain” to “the most severe pain imaginable.” Participants were asked to mark their perceived pain intensity on the line. Subsequently, the distance from the “no pain” endpoint to the marked location was measured using a millimeter ruler, and pain intensity was quantified in centimeters (cm) [18].

### Assessment of quality of life

The assessment of quality of life of the adult individuals with IS employed the Scoliosis Research Society-22 (SRS-22), a specialized 22-item questionnaire designed to evaluate scoliosis-specific quality of life. The SRS-22 encompasses domains such as pain, body image/appearance, functional capacity, mental health, and treatment satisfaction. Responses to each question are rated on a scale ranging from 0 (worst) to 5 (best), with higher scores reflecting a higher quality of life [19].

### Assessment of cosmetic deformity perception

The Turkish version of the Walter Reed Visual Assessment Scale (WRVAS) was used to evaluate the patients’ perception of cosmetic deformity [20]. This scale comprises seven items, each featuring figures representing various aspects of spinal deformity: thoracic deformity, shoulder asymmetry, spinal curvature, lumbar protrusion, scapular asymmetry, and rib protrusion. Each figure in the items is scored, ranging from 1 point for ‘no deformity’ to 5 points for ‘severe deformity’. A higher score indicates a perception of a more severe deformity [21].

### Assessment of curvature severity

The Cobb method, which is accepted as the gold standard measurement method, was used to evaluate the severity of the scoliosis curve in patients. The Cobb angle was measured on a standard standing posteroanterior spine radiograph [22].

### Assessment of trunk rotation angle

The thoracic TRA was evaluated using a Bunnell scoliometer in the Adam’s forward bending test position, with the scoliometer positioned perpendicular to the axial axis of the spine and aligned with the spinous process of the apical vertebra. The rotation angle was then recorded in degrees [23].

### Assessment of disability

The Turkish version of the Oswestry Disability Index (ODI) was used to assess functional disability associated with scoliosis [24]. The ODI stands out as the predominant disease-specific patient-reported outcome questionnaire for assessing functional disability related to back pain. Within the index, various daily life activities are assessed from diverse angles. A high score signifies the utmost degree of functional disability concerning back pain. A high percentage value obtained from the index indicates high disability [25].

### Assessment of spinal mobility

Spinal mobility was measured with a computerized portable electromechanical device (The Spinal Mouse System, Idiag, Fehraltorf, Switzerland) which has been proven to be reliable and valid. Measurements were made between the spinous process of C7 and the apex of the anal fold (approximately S3). The maximum degrees of flexion-extension in the sagittal plane (SP) and maximum degrees of right-left lateral flexion in the frontal plane (FP) were measured and recorded [26].

### Statistical methods

Initially, the descriptive statistics of the employed data have been summarised as frequencies and percentages for qualitative values, and as mean, standard deviation, minimum and maximum values for quantitative variables. The correlations between the variables that were found to be normally distributed using visual and analytical methods were evaluated using Pearson correlation analysis. A correlation coefficient below 0.30 was interpreted as indicating low correlation, between 0.30 and 0.60 as moderate correlation, and above 0.60 as high correlation [27].

A number of advanced regression models including forward regression, backward regression, stepwise regression, ridge regression [28], lasso regression [29], and elastic net regression [30] have been employed to predict the VAS scores of individuals with IS. In order to detect whether there was multicollinearity, tolerance and variance inflation factor (VIF) values were calculated for each independent variables SRS-22, WRVAS, Cobb angle, TRA, ODI, total FP motion, and total SP motion, which were normally distributed. For all variables, tolerance values greater than 0.10 and VIF values less than 10 were accepted as having no multicollinearity [31]. The regression analysis process can be briefly outlined as follows:

- The data has been split into 75% for training and the rest for testing data. The models have been developed using training data and the performance metrics have been calculated on the test data.
- As the cross-validation (CV) method, five-times repeated five-folds CV has been implemented.
- Thirty-valued grid space for lambda parameter has been investigated as the parameter space for the ridge, lasso and elastic net regression models, and optimum parameterized models have been found following CV.
- The root mean squared error (RMSE), mean absolute error (MAE) and $\:{R}^{2}$ (the coefficient of determination) have been considered as performance criteria which are calculated as follows:

$$\:RMSE=\sqrt{\frac{1}{{n}}\sum\:_{{i}=1}^{{n}}{\left({{t}}_{{i}}-{{y}}_{{i}}\right)}^{2}}$$

$$\:MAE=\frac{1}{n}\sum\:_{i=1}^{n}\left|{t}_{i}-{y}_{i}\right|$$

$$\:{R^2} = 1 - \frac{{\sum {\:_{i = 1}^n} {{\left( {{t_i} - {y_i}} \right)}^2}}}{{\sum {\:_{i = 1}^n} {{\left( {{t_i} - \bar t} \right)}^2}}}$$

where the target value $\:\left({t}_{i}\right)$ and the predicted value $\:\left({y}_{i}\right)$ by the model. When interpreting these metrics, the lower value of RMSE or MAE indicates a better model, higher values of $\:{R}^{2}$ provides a more explainable model. The coefficient estimates have been obtained and interpreted utilizing the optimum regression model. The agreement between the model predictions and actual VAS scores has then been supported through a scatter plot. The analysis of the study has been conducted with the R software (v.4.3.2), some R packages including caret [32], tidymodels [33] and the statistical significance value has been taken as 0.05 in the analysis process.

## Results

In total, 82 adults with IS were referred for evaluation. Of these, 24 individuals were excluded from the study who did not have a Lenke type 1 curve (*n* = 11), had undergone conservative scoliosis treatment within the last year (*n* = 8), and had a history of previous spine surgery or trauma (*n* = 5). As a result, the study was completed with 58 adult individuals with IS. The summary statistics of the study and the correlation levels between variables are presented in Tables 1 and 2, respectively. Based on the results in Table 1, the ages of the individuals participated in the study is 29.31 ± 4.53, the mean VAS score is $\:6.05$ and the standard deviation is $\:1.26$. The participants, 39% of whom are women, are mostly individuals with no exercise behavior (86.21%).

**Table 1:** The descriptive statistics of the dataset used in this study

	Mean ± SD	Minimum	Maximum
Age (years)	29.31 ± 4.53	21.00	39.00
BMI (kg/m^2^)	25.38 ± 2.37	20.20	31.13
SRS-22 (point)	3.11 ± 0.56	2.00	4.00
WRVAS (point)	18.38 ± 3.76	12.00	25.00
Cobb angle (º)	50.98 ± 7.75	37.00	55.00
Trunk rotation angle (º)	25.55 ± 4.60	16.00	30.00
Oswestry Disability Index (%)	33.38 ± 8.05	16.00	46.00
Total FP motion (º)	52.07 ± 11.85	27.00	76.00
Total SP motion (º)	94.26 ± 11.52	69.00	116.00
VAS (cm)	6.05 ± 1.26	3.00	9.00

		Count	Percentage
Gender	Female	39	67.24
	Male	19	32.76
Dominant side	Right	53	91.38
	Left	5	8.62
Curve type	Right thoracic	38	65.52
	Left thoracic	20	34.48
Exercise behaviour	Yes	8	13.79
	No	50	86.21

SD: Standart deviation, BMI: Body mass index, SRS-22: Scoliosis Research Society-22, WRVAS: Walter Reed Visual Assessment Scale, FP: Frontal plane, SP: Sagittal plane, VAS: Visual Analogue Scale

The results of the correlation analysis showed that there were high positive correlations between.

VAS score and SRS-22 score, WRVAS score, Cobb angle, TRA, and ODI score, while there were high negative correlations between VAS score and total FP and SP motions (Table 2).

**Table 2:** Results of the correlation analysis between the VAS score and other variables

Variables	SRS-22	WRVAS	Cobb angle	TRA	ODI	Total FP motion	Total SP motion
VAS	r (p)	r (p)	r (p)	r (p)	r (p)	r (p)	r (p)
	0.75 (0.012)	0.91 (0.006)	0.92 (0.017)	0.85 (0.003)	0.91 (0.009)	-0.88 (0.011)	-0.89 (0.016)

VAS: Visual Analogue Scale, SRS-22: Scoliosis Research Society-22, WRVAS: Walter Reed Visual Assessment Scale, TRA: Trunk rotation angle, ODI: Oswestry Disability Index, FP: Frontal plane, SP: Sagittal plane

Tolerance values for all independent variables were found between 0.63 and 0.92 and were higher than 0.10. VIF values were found between 1.55 and 2.36 and were less than 10. These findings confirmed the absence of multicollinearity. The performance metrics of six regression models are reported in Table 3. It is clear from Table 3 that the Lasso regression outperforms the rest of the models in terms of all three criteria $\:(RMSE=0.247,\:MAE=0.216\:and\:{R}^{2}=0.959)$. The Lasso regression is able to explain 95.9% of the variation in VAS score (Table 3).

**Table 3:** The comparison results of model performances with respect to three different metric scores

Metric/ Model	Forward regression	Backward regression	Stepwise regression	Ridge regression	Lasso regression	Elastic Net regression
RMSE	0.341	0.415	0.341	0.252	**0.247**	0.249
MAE	0.280	0.348	0.281	0.331	**0.216**	0.247
R^2^	0.924	0.871	0.925	0.947	**0.959**	0.958
Optimum parameters				lambda = 0.405	lambda = 0.413	lambda = 0.055

RMSE: Root mean squared error, MAE: Mean absolute error, R^2^: the coefficient of determination

The coefficient values of these models considered in the study with their optimum parameters on the test data are listed in Table 4. The following conclusions can be inferred by analyzing the coefficients given in Table 4.

i. The most restrictive models are forward and stepwise regressions, while the most flexible models are ridge and elastic net regressions. The forward and stepwise regressions retained the least number of variables in the model and only Cobb angle, ODI and total SP motion measurements found to be significant.
ii. The ODI and total SP motion measurements are significant and beneficial across the all models.

The mathematical model derived for Lasso regression, as the best model, can be expressed as follows:

$$\begin{array}{l}{\rm{VAS}}\:{\rm{Score}} = 5.270 + 0.122 \times \:{\rm{Cobb}}\:{\rm{angle}} + 0.021 \times \:{\rm{SRS}} - 22\\+ 0.033 \times \:{\rm{Trunk}}\:{\rm{rotation}}\:{\rm{angle}} + 0.033 \times \:{\rm{WRVAS}}\\+ 0.032 \times \:{\rm{Oswestry}}\:{\rm{Disability}}\:{\rm{Index}}\: - 0.039 \times \:{\rm{Total}}\:{\rm{SP}}\:{\rm{motion}}\:\end{array}$$

**Fig. 1:**
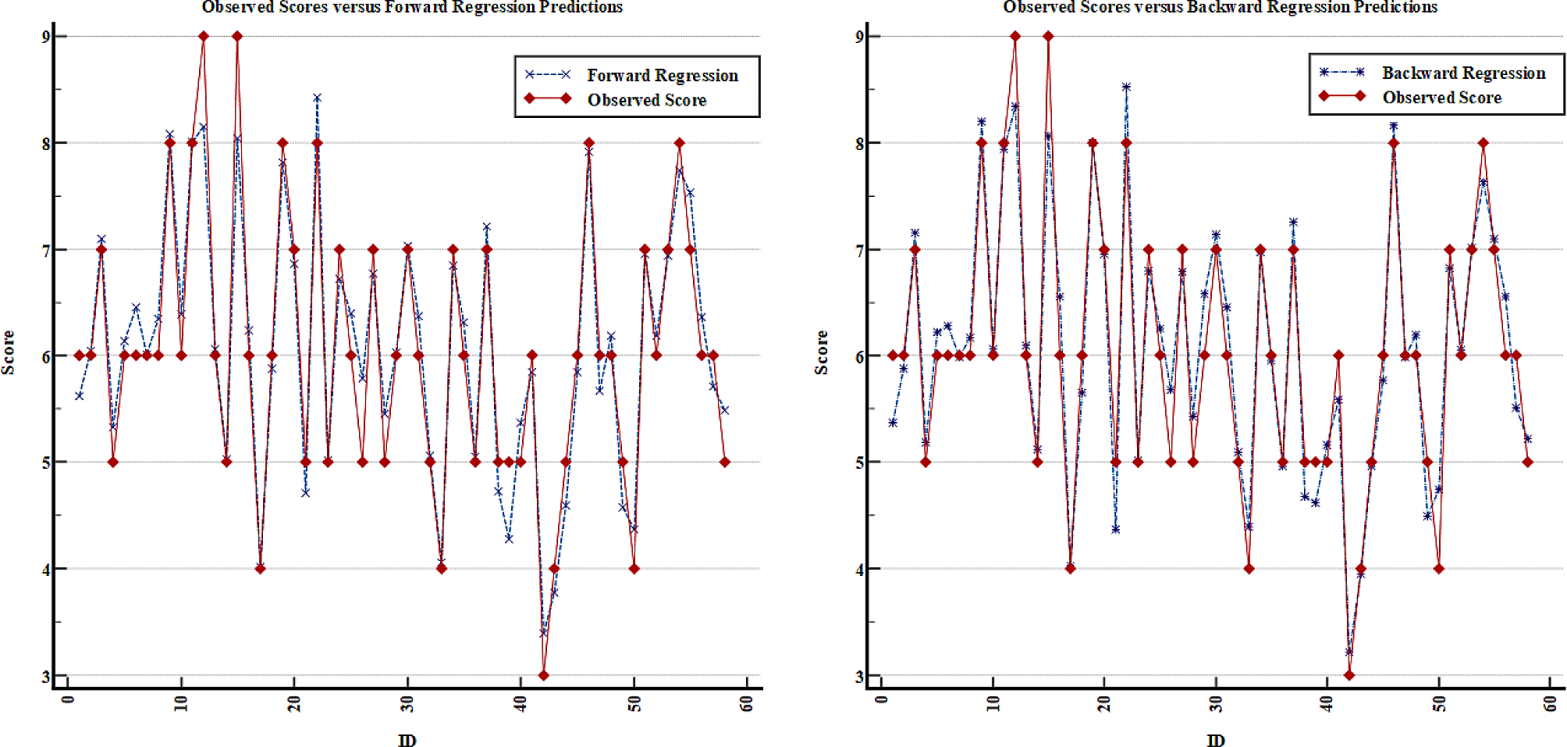
The comparison of observed and predicted VAS scores based on forward and backward regression models

**Fig. 2:**
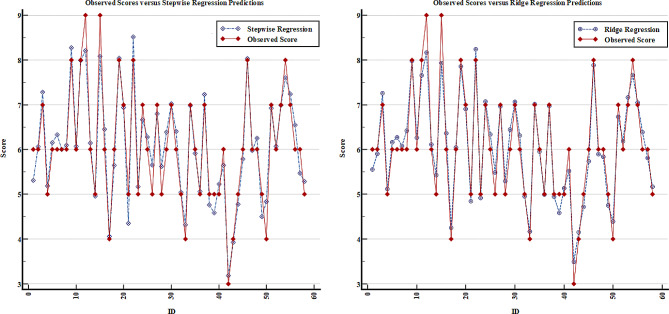
The comparison of observed and predicted VAS scores based on stepwise and ridge regression models

**Fig. 3:**
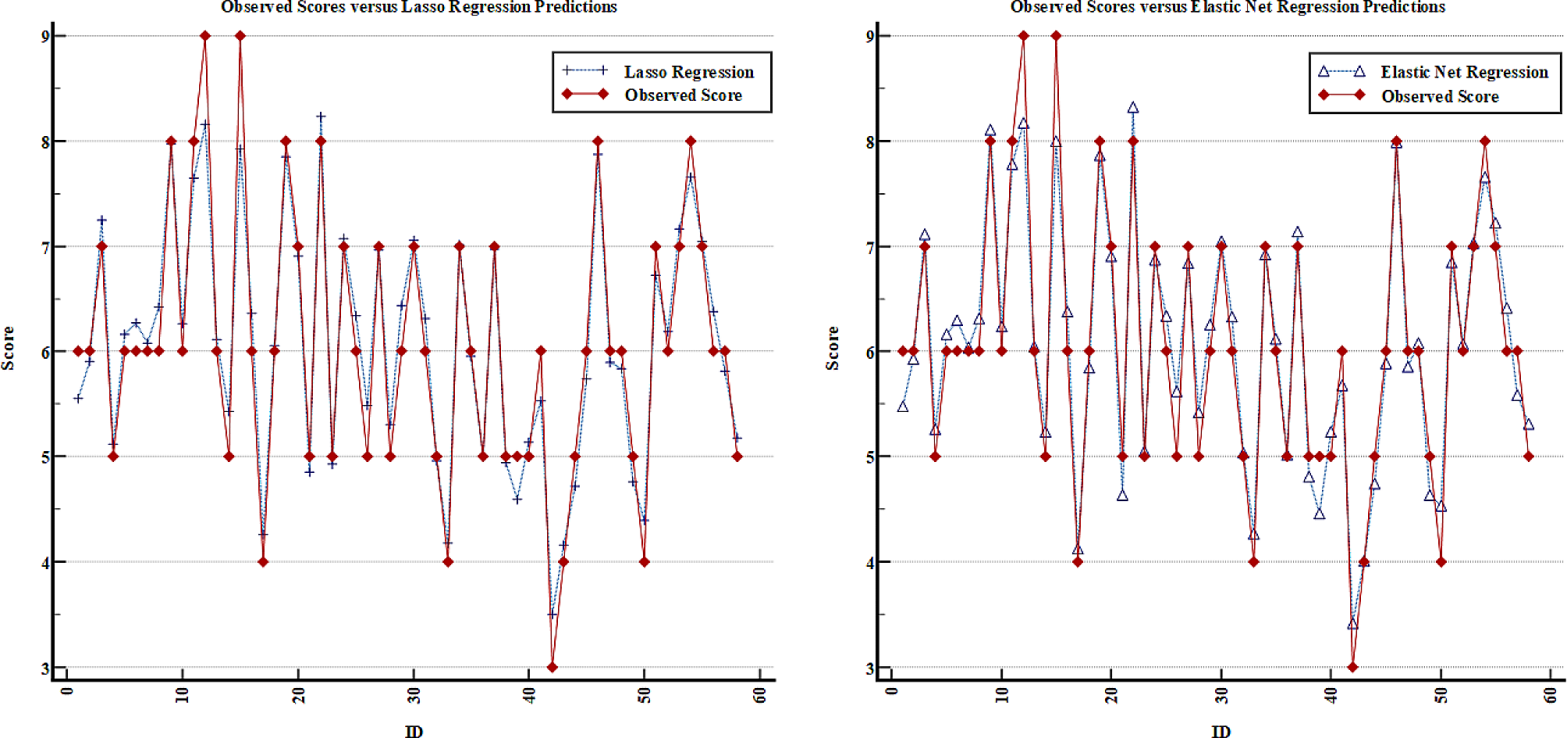
The comparison of observed and predicted VAS scores based on Lasso and elastic net regression models

When the model coefficients are analyzed, it can be said that a one-unit increase in the Cobb angle increases the VAS score by 0.122 units on average, holding other measurements as constant. Likewise, the mean VAS score tends to increase when the total SP motion measurement decreases or the TRA, WRVAS, ODI, and SRS-22 scores increase (Table 4). The scatter plots of each model are given in Figs. 1, 2 and 3 to assess the fit between the observed VAS value and model predictions. Based on these figures, it is clear that the Lasso regression predicts the measured VAS score better than the other models and the difference between them is relatively small (Fig. 3).

**Table 4:** The coefficient estimates derived based on each regression model by using the test data

Model	Forward Regression	Backward Regression
Coefficients	*Intercept = 5.355* Cobb angle = 0.061 Total SP motion= -0.042 ODI = 0.0390	*Intercept = 6.435* TRA = 0.065 ODI = 0.046 Total SP motion= -0.043 WRVAS = 0.041

Model	Stepwise Regression	Ridge Regression
Coefficients	*Intercept = 5.356* Cobb angle = 0.062 Total SP motion= -0.0425 ODI = 0.039	*Intercept = 3.537* Cobb angle = 0.302 TRA = 0.096 WRVAS = 0.038 Exercise behaviour (no) = 0.037 ODI = 0.026 SRS-22 = 0.025 Total SP motion= -0.023 Total FP motion= -0.010 Age = 0.008

Model	Lasso Regression	Elastic Net Regression
Coefficients	*Intercept = 5.270* Cobb angle = 0.122 Total SP motion= -0.039 TRA = 0.033 WRVAS = 0.033 ODI = 0.032 SRS-22 = 0.021	*Intercept = 3.534* Cobb angle = 0.301 TRA = 0.096 WRVAS = 0.039 Exercise behaviour (no) = 0.037 ODI = 0.026 SRS-22 = 0.025 Total SP motion= -0.023 Total FP motion= -0.011 Age = 0.008

SRS-22: Scoliosis Research Society-22, WRVAS: Walter Reed Visual Assessment Scale, TRA: Trunk rotation angle, ODI: Oswestry Disability Index, FP: Frontal plane, SP: Sagittal plane

## Discussion

To the best of our knowledge, this first study examining independent predictors of pain intensity in adult IS revealed that the Lasso regression model exhibited the highest performance across the evaluated criteria, elucidating 95.9% of the variation in VAS scores. As per this model, the leading determinants of pain severity in adult IS, ranked by significance, encompassed curvature severity, spinal mobility, TRA, perception of cosmetic deformity, disability related to the back, and quality of life.

Adult scoliosis represents a significant condition impacting the aging spine. Given the trend towards an aging population, it is crucial to ascertain the factors influencing the manifestation of symptoms, such as pain, in patients diagnosed with scoliosis [34]. Decreased physical capacity, respiratory problems, back pain, decreased spinal mobility, and cosmetic problems are the main problems suffered by patients with unsurgically treated adult IS [35].

Cobb angle is the most widely utilized radiologic parameter to assess the severity and progression of deformity in individuals with scoliosis, as well as the effectiveness of treatments [36]. In the literature, several studies have explored the correlation between Cobb angle and pain intensity. Among these, a study on adult individuals with lumbar scoliosis revealed a relationship between pain intensity and radiologic parameters such as Cobb angle and TRA, and it was proposed that curve severity is a factor that increases pain intensity [16]. Notably, Fortin et al. [37] established significant correlations, indicating that lower Cobb angles were linked to reduced pain severity in patients diagnosed with AIS. Highlighting that pain in scoliosis primarily stems from the concavity of the curves, encompassing discogenic, radicular, and facet joint origins, Jackson et al. [38] observed a positive correlation between pain intensity and the degree of scoliotic curvature. It has been documented that the consideration of vertebral axial rotation is important in assessing the severity and prognosis of scoliosis. Ferrero et al. [39] reported a significant association between vertebral axial rotation and pain intensity in adults with scoliosis, noting that pain intensity notably increased in patients exhibiting more than 10° of vertebral axial rotation. However, in another study, Fekete et al. [15] reported that there was no relationship between pain intensity and Cobb angle in adults with IS. In parallel with the results of the majority of the studies in the literature [38, 39], this study revealed that there are strong relationships between Cobb angle and TRA and pain intensity and that Cobb angle and TRA are two important determinants of pain intensity according to the best performing regression model. These results suggest that imbalance resulting from compression and tension stresses due to asymmetric loading in the discs, facet joints, muscles, and ligaments in the spine may have caused pain.

Patients with scoliosis have reduced spinal mobility and flexibility due to structural deformity of the spine, which can deteriorate over time. The negative effects of loss of spinal mobility may include more back pain, trunk rigidity, and a lower quality of life [40]. Deviren et al. [40] assessed axial pain and flexibility among patients with IS, identifying a noteworthy correlation between flexibility and pain intensity. Danielsson et al. [41] assessed spinal mobility, muscle endurance, back pain, and functional outcomes in individuals with AIS two decades following brace treatment, revealing significant correlations between spinal mobility and the severity of back pain. In the present study, total SP motion, one of the parameters used to determine spinal mobility, was found to be the second most important predictor of pain intensity according to the best performing regression model. Also, consistent with the literature, strong associations were observed between spinal mobility and pain intensity. Scoliosis, which decreases the flexibility and mobility of the spine and adversely affects its alignment, may have impaired the postural reflex mechanism and involvement of paraspinal muscles, leading to the onset and exacerbation of pain. However, given the quite limited number of studies examining the relationships between spinal mobility and pain intensity in adult IS, it is recommended that these relationships be investigated more extensively in future studies.

As per SOSORT guidelines, examining the cosmetic deformity perception among individuals with scoliosis holds significance in the development of assessment and treatment protocols [2]. Pineda et al. [42] documented that the WRVAS scale exhibits sensitivity to cosmetic alterations arising from either deterioration or improvement in the scoliotic deformity. Relatively few studies on scoliosis, about 5%, include an evaluation of cosmetic appearance [2]. Savvides et al. [43] reported that individuals with IS were more concerned with their cosmetic appearance than individuals without scoliosis. Studies have shown that patients with thoracic curvature greater than 40° and trunk rotation greater than 20° have a negative self-image regarding their cosmetic appearance [42, 44, 45]. Pineda et al. [42] found a moderately significant relationship between the pain subdomain of the SRS-22 scale and the WRVAS score in individuals with IS. In another study conducted by Makino et al. [46] to determine possible risk factors for back pain in individuals with AIS, it was proposed that poor self-image, determined according to the SRS-22 scale, may be a risk factor for back pain. In the current study, a high correlation was observed between pain intensity and the cosmetic deformity perception score measured by WRVAS, in agreement with these studies. Another notable finding of this study was that WRVAS score may be an important predictive factor for pain intensity. This result suggests that cosmetic deformity perception may be a predictor of pain intensity in adult individuals with IS. Poor cosmetic deformity perception may have negatively affected the psychological state of the individuals, altering their pain perception processes and causing them to experience more pain.

In general, individuals with scoliosis typically do not encounter significant functional impairment that would substantially impede their ability to carry out daily activities [47]. Nonetheless, adolescents and adults with scoliosis exhibit a markedly higher incidence of back pain compared to the general population, with the risk of experiencing pain, functional impairment, and disability escalating with age [5, 34, 48]. Studies have reported that back pain may be associated with disability in adult individuals with IS [49]. In alignment with these findings, Jeon et al. [50] identified a strong correlation between the severity of back pain, as assessed by VAS, and the ODI score among adult individuals with scoliosis. On the other hand, Smith et al. [51] reported that the majority of individuals with adult scoliosis had significant back pain and disability, and that more back pain in these individuals was associated with more disability and poorer health status. In the present study, a robust association was detected between pain intensity and ODI score in support of the literature, and it was also revealed that ODI score was an important possible predictor of pain intensity according to the best-performing Lasso regression model. Considering these findings in the literature and in the present study, it can be said that disability level is a significant determinant of pain severity in adults IS.

In this study, the strong correlation between the level of quality of life assessed according to the SRS-22 scale and pain severity was consistent with the literature. Another interesting finding of the study was that the quality of life parameter was a possible predictor of pain intensity. Studies point to possible relationships between quality of life and pain intensity in individuals with scoliosis. Makino et al. [13] reported that low back pain may be associated with poor quality of life in individuals with AIS. Similarly, in another study conducted in individuals with IS, Lia et al. [52] documented significant relationships between the pain subscale score of the SRS-22 scale and the Short Form-36 quality of life questionnaire score. In another study conducted in adult individuals with scoliosis, moderate significant relationships were found between pain intensity and Short Form-36 quality of life questionnaire scores [50]. Makino et al. [46] suggested that self-image and mental health sub-parameters of the SRS-22 may be possible risk factors for chronic back pain in their study in individuals with AIS, and they also reported that postural changes in spinal alignment and psychological problems may play important roles in the occurrence of back pain. In the current study, quality of life was found to be a possible influential factor in pain in adult individuals with IS, suggesting that negative impact on quality of life may have effects on the processes of pain occurrence and perception, including psychological status. However, it may be useful to investigate these possible mechanisms in further studies to reach a more definitive conclusion. To the best of our knowledge, no study has investigated the possible effects of quality of life on pain in adult individuals with IS, limiting our ability to adequately discuss our findings with the literature. To the best of the authors’ knowledge, this is the first study to suggest that quality of life may be a predictor of pain severity in individuals with adult IS.

This study has some limitations. The first of these is that only individuals with non-degenerative IS with Lenke type 1 curvature were included in the study. Therefore, generalization of the results of this study to individuals with other curve types or degenerative adult scoliosis may not be appropriate. The second is that psychological variables were not included in the methodology when the study was planned. Considering that psychological problems may be factors that may affect pain, further studies are needed to examine the determinants of pain including psychological factors in adult individuals with different curve types and degenerative scoliosis. Another limitation is that individuals’ quality of life was assessed with SRS-22. It has been reported that SRS-22 may have a ceiling effect in the assessment of quality of life in IS [53]. Therefore, in future studies, it may be more appropriate to assess quality of life in adult individuals with IS using the Brace Questionnaire or the Italian Spine Youth Quality of Life questionnaire.

## Conclusion

In this study, which examined for the first time the determinants of pain severity in individuals with non-surgically treated Lenke type 1 adult IS, strong associations were found between pain severity with curvature severity, spinal mobility, TRA, perception of cosmetic deformity, disability, and quality of life. Regression analysis showed that Lasso regression was the best model according to performance criteria. According to this model, the primary determinants of pain severity in adult IS, listed in order of significance, include curvature severity, spinal mobility, TRA, perception of cosmetic deformity, back-related disability, and quality of life. Given that pain substantially impacts the daily activities and quality of life of individuals with IS, it is beneficial to consider the mentioned possible factors that affect and determine pain when establishing assessment and treatment programs.

## Data Availability

No datasets were generated or analysed during the current study.
